# A 7-Step Guideline for Qualitative Synthesis and Meta-Analysis of Observational Studies in Health Sciences

**DOI:** 10.3389/phrs.2023.1605454

**Published:** 2023-05-16

**Authors:** Marija Glisic, Peter Francis Raguindin, Armin Gemperli, Petek Eylul Taneri, Dante Jr. Salvador, Trudy Voortman, Pedro Marques Vidal, Stefania I. Papatheodorou, Setor K. Kunutsor, Arjola Bano, John P. A. Ioannidis, Taulant Muka

**Affiliations:** ^1^ Institute of Social and Preventive Medicine, University of Bern, Bern, Switzerland; ^2^ Swiss Paraplegic Research, Nottwil, Switzerland; ^3^ Graduate School for Health Sciences, University of Bern, Bern, Switzerland; ^4^ Faculty of Health Science and Medicine, University of Lucerne, Lucerne, Switzerland; ^5^ Institute of Primary and Community Care, University of Lucerne, Lucerne, Switzerland; ^6^ HRB-Trials Methodology Research Network, National University of Ireland, Galway, Ireland; ^7^ Department of Epidemiology, Erasmus MC, University Medical Center, Rotterdam, Netherlands; ^8^ Division of Human Nutrition and Health, Wageningen University and Research, Wageningen, Netherlands; ^9^ Department of Medicine, Internal Medicine, Lausanne University Hospital (CHUV) and University of Lausanne, Lausanne, Switzerland; ^10^ Department of Epidemiology, Harvard TH Chan School of Public Health, Boston, MA, United States; ^11^ Diabetes Research Centre, University of Leicester, Leicester General Hospital, Leicester, United Kingdom; ^12^ Translational Health Sciences, Bristol Medical School, University of Bristol, Southmead Hospital, Bristol, United Kingdom; ^13^ Department of Cardiology, Inselspital, Bern University Hospital, University of Bern, Bern, Switzerland; ^14^ Stanford Prevention Research Center, Department of Medicine, Stanford University School of Medicine, Stanford, CA, United States; ^15^ Department of Epidemiology and Population Health, Stanford University School of Medicine, Stanford, CA, United States; ^16^ Department of Statistics, Stanford University, Stanford, CA, United States; ^17^ Meta-Research Innovation Center at Stanford (METRICS), Stanford University, Stanford, CA, United States; ^18^ Epistudia, Bern, Switzerland

**Keywords:** cross-sectional studies, observational study, evidence-based approach, cohort studies, systematic review and meta-analysis

## Abstract

**Objectives:** To provide a step-by-step, easy-to-understand, practical guide for systematic review and meta-analysis of observational studies.

**Methods:** A multidisciplinary team of researchers with extensive experience in observational studies and systematic review and meta-analysis was established. Previous guidelines in evidence synthesis were considered.

**Results:** There is inherent variability in observational study design, population, and analysis, making evidence synthesis challenging. We provided a framework and discussed basic meta-analysis concepts to assist reviewers in making informed decisions. We also explained several statistical tools for dealing with heterogeneity, probing for bias, and interpreting findings. Finally, we briefly discussed issues and caveats for translating results into clinical and public health recommendations. Our guideline complements “A 24-step guide on how to design, conduct, and successfully publish a systematic review and meta-analysis in medical research” and addresses peculiarities for observational studies previously unexplored.

**Conclusion:** We provided 7 steps to synthesize evidence from observational studies. We encourage medical and public health practitioners who answer important questions to systematically integrate evidence from observational studies and contribute evidence-based decision-making in health sciences.

## Introduction

Observational studies are more common than experimental studies (1, 2). Moreover, many systematic reviews and meta-analyses (SRMA) integrate evidence from observational studies. When undertaking synthesis and MA, it is crucial to understand properties, methodologies, and limitations among different observational study designs and association estimates derived from these studies. Different study designs influence variability in results among studies, and thus heterogeneity and conclusions (Supplementary Material S1). Specific study type considerations and methodological features include (among others): study participant selection and study sample representation; measurement and characterization methods for exposure and extent of information bias; potential confounders and outcomes; design-specific contributions leading to bias; and methods used to analyze the data. Furthermore, observational studies may have a wider array of selective reporting biases than randomized trials. Most observational studies are unregistered, and typically more degrees of analytical flexibility and choice of analyses report such designs compared with randomized trials, leading to more variable results and potential bias (3). These methodological components influence study design suitability and result in trustworthiness for SRMA. Indeed, evidence shows that MAs of observational studies often suffer methodologically (1), and despite statistical or other summary result significance, many observational studies demonstrate low credibility (2). Observational data often complement evidence from randomized controlled trials (RCTs) when shaping public health and clinical guidelines and recommendations. Yet, observational data for informing public health and clinical decision-making are inconsistently available in SRMAs. Therefore, we provide concise guidance for combining results in a MA of observational studies.

## Methods

The current guideline was developed by a multidisciplinary team of researchers with extensive experience in SRMAs. The guide extends a previous guideline (4) and provides further recommendations for synthesizing and pooling results from observational data. For this, we considered previous guidances for SRMA of observational studies (5–7), and acknowledged several contentious points concerning optimal methods for MA of observational studies (8). We explicitly address such uncertainties and offer definitive recommendations for uncontested best practices. Finally, we offer guidance relevant to diverse types of observational data subject to SRMA. However, the range of observational data types, such as adverse drug events, genetic associations, effectiveness studies, nutritional associations, air pollution, and prevalence studies, is broad. Therefore, proper evidence synthesis requires knowledge of best SR practices and field-specific subject matter.

## Results

### Step-by-Step Guide

**FIGURE 1:**
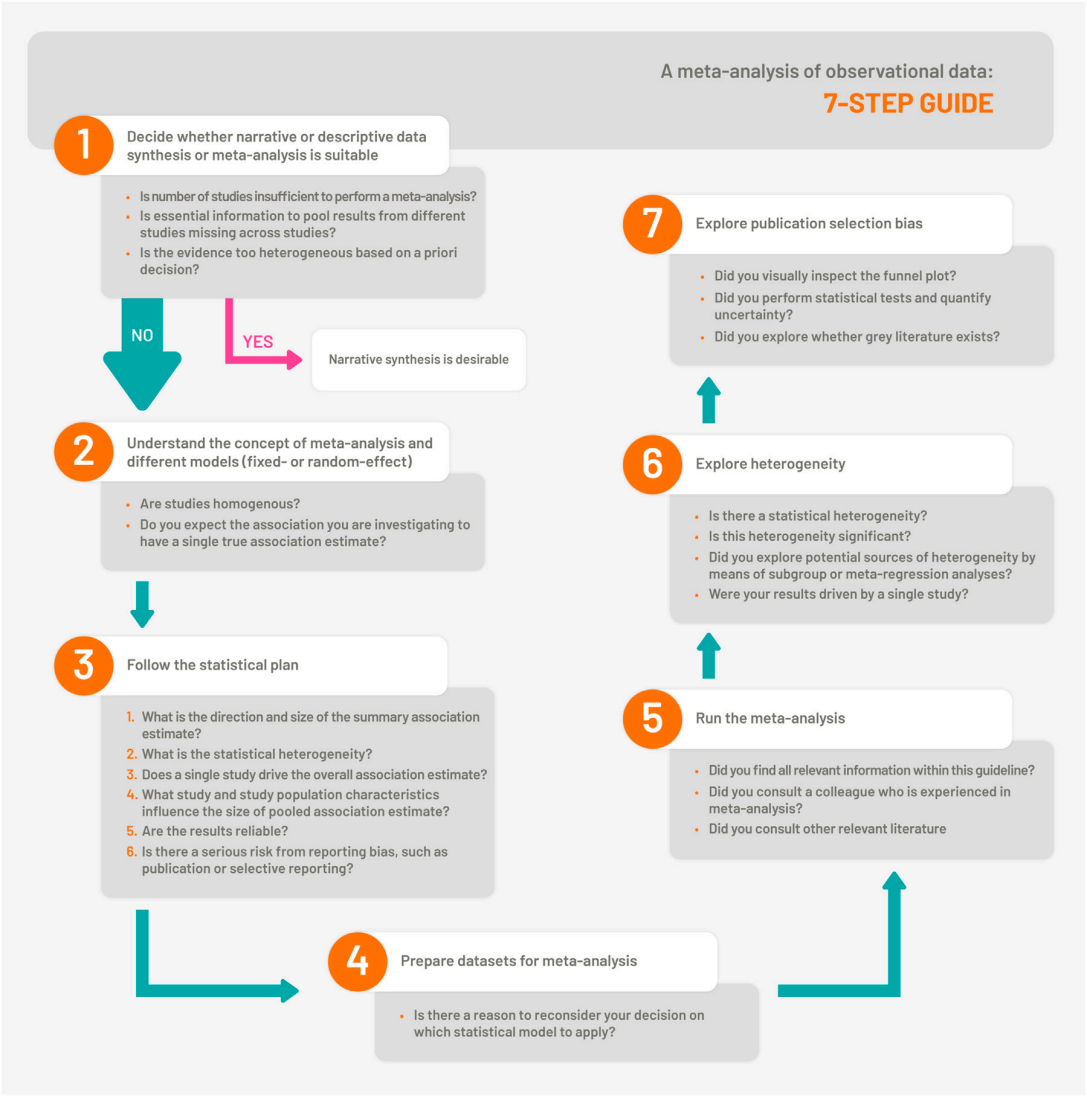
The 7-Step Guide which illustrates the steps for synthesis and meta-analysis of observational studies (Bern, Switzerland. 2023).

The overall step-by-step guidance is visualized in Figure 1.

#### Step 1. Decide Whether Narrative or Descriptive Data Synthesis or Meta-Analysis is Suitable

**FIGURE 2:**
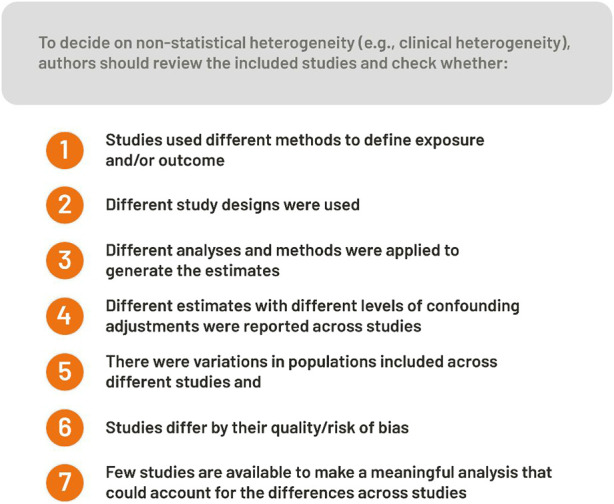
Factors to consider on whether to perform a meta-analysis or not (Bern, Switzerland. 2023).

When summarizing evidence from observational studies, narrative or descriptive data synthesis is desirable when: a) the number of studies is insufficient to perform MA; b) essential information to combine results from different studies is missing across studies; or c) the evidence is judged as too heterogeneous, such as clinical heterogeneity, based on *a priori* decision. We provide tips for determining when clinical heterogeneity is too high in Figure 2. We caution early, careful thinking and decision-making about handling complex patterns of bias in available evidence and pre-specified protocols. Otherwise, observed results can drive included study choices prematurely.

a. **How many studies are sufficient for MA?** MA is possible if association estimates from two studies are available. However, deciding to perform a MA (9)—see Step 2 for choosing statistical models—is influenced by differences in study design, exposure, adjustment, outcome assessment, study population, risk of bias, and other methodological features across studies.
b. **What information is essential for MA?** To combine study results, measurements of association estimates from individual studies and standard errors or 95% confidence intervals (CIs) of the estimate are needed. For details about combining different estimates and information needed, see Step 3 and Supplementary Table S1. We suggest contacting the corresponding authors for missing essential information.
c. **When is heterogeneity too large?** Without widely accepted, automated quantitative measures to grade it, determining whether clinical or methodological heterogeneity is too high is subjective. Heterogeneity can result from methodological differences, such as different study designs, analytical assessments of exposures/outcomes, or variations among populations across different studies; it requires restricting MA based on study population, design, exposure, or outcome characteristics. To see how statistical heterogeneity is explored quantitatively using I^2^ or Cochran Q statistics, see Step 6. Deciding to perform MA should not be based on statistical heterogeneity.
d. **Do “study quality” and methodological rigor determine whether to meta-analyze the evidence?** “Study quality” is a complex term; it involves assessing methodological rigor (what was done) and completeness or accuracy of reporting (what is reported to have been done) within individual studies. Established and validated risk of bias tools can evaluate individual studies included in SR, which can inform the synthesis and interpretation of results. Poor methodological rigor and incomplete or inaccurate reporting of individual studies can bias synthesized results and limit MA interpretation and generalizability. Thus, potential biases across included studies should be systematically assessed. Various tools and scales can be used to assess methodological rigor and reporting. We summarize these scales in Supplementary Material S2, Supplementary Table S2 **.**
e. Does the study design determine whether to meta-analyze the evidence?

Including all study designs in SRs reduces subjective interpretations of potential biases and inappropriate study exclusions (6); however, the decision to meta-analyze results across all study designs depends on research questions. For example, cross-sectional designs are likely inappropriate for research questions dealing with temporality but could be used to summarize prevalence estimates of diseases. If different study designs are included in SRs, address heterogeneity by study design in the MA step and perform subgroup analyses by study design otherwise, misleading results can follow (10).

Overall, when deciding to remove studies from MA due to poor methodology, it is crucial to evaluate the extent of bias across available evidence (i.e., bias in single or multiple studies). If all available studies provide biased estimates, MA simply provide a composite of these errors with low-reliability results perpetuating these biases. If only a proportion of studies are biased and subsequently included in MA, stratification by methodological features may be a solution. However, even with enough studies in the synthesis to perform subgroup analysis, it is informative only. More details are provided in Steps 6 and 7.

After carefully considering Step 1 items a–e, if MA is not feasible or meaningful, summarize findings qualitatively with narrative or descriptive data synthesis. Descriptive data synthesis is not necessarily worse or lower quality compared with MA. Depending on the number of included studies and methodological differences across studies in a descriptive synthesis, writing a narrative data summary can prove more difficult compared with MA. In Table 1, we provide insights for simplifying the process of descriptive data synthesis. We use examples, such as grouping studies and presenting data from previously published SRs summarizing evidence without MA (12–15).

**TABLE 1:** Steps to consider when conducting a narrative summary of evidence (Bern, Switzerland. 2023).

Step 1	Group studies	Choose an appropriate grouping rationale
		A. PECO [population (male only participants vs. mixed population; healthy vs. individuals with comorbidities; animal vs. human evidence)], exposure/comparison and outcome (reported on continuous vs. dichotomized scale)
		B. Study design (cross-sectional vs. longitudinal studies)
		C. Risk of bias (low quality vs. moderate or high-quality evidence)
		D. Association estimates: consider type (beta coefficients, risk ratios, odds ratios, hazard ratios, etc.) and direction of association (higher risk in exposed population vs. no association). To accurately interpret *p-values* and 95% confidence intervals, identify and understand the direction of associations
Step 2	Follow the same synthesis consistently	A. Create additional tables using study groupings to find patterns among studies. For example, provide separate tables for cross-sectional and cohort studies.
		B. Convert association estimates if possible. For comparison among studies, convert odds ratios to standardized mean differences
		C. Present most interesting findings using graphical methods, such as arrows indicating increased or decreased risk between groups
		D. If meta-analysis is not possible, use the data extraction sheet to conduct minimal statistical analyses. For example, calculate total numbers of study participants, mean age, mean number of male participants, or other relevant study population, exposure, or outcome characteristic
Step 3	Report findings clearly	A. Use appropriate language
		B. Keep reporting style uniform across results section. For instance, if studies are grouped, start with a paragraph explaining grouping variables
		C. Provide summary tables and/or figures to support findings reported in results section
Step 4	Discuss findings objectively	Summarizing what best reflects reviewed evidence can be challenging
		A. Report based on grouping parameters from Step 1. Graphical summaries support interpreting findings (especially when analyzing many methodologically different studies)
		B. Discuss methodological strengths and limitations of reviewed evidence. For example, levels of adjustment across studies, heterogeneity that precluded quantitative synthesis, or risks of bias
		C. Identify literature gaps and provide directions for future research

We suggest providing graphical summaries of important findings, especially when tables and figures amass complex, convoluted information [e.g., second figure of SR by Oliver-Williams et al. (12)]. If MA is inappropriate, another graphical option is a forest plot without the overall association estimate^15^—a display that promotes reader insights on association estimate size and 95% CIs across studies. We also recommend synthesis without MA (SWiM) reporting guidelines (11) to assist in reporting findings from SRs without MA. Finally, although narrative synthesis of evidence is the default choice when performing an SR of qualitative research, it extends beyond the scope of our guidelines. Several guidelines exist on SR of qualitative studies (16, 17).

#### Step 2. Understand the Concept of Meta-Analysis and Different Models

Combining results from different observational studies can lead to more comprehensive evaluations of evidence, greater external validity, and higher precision due to larger sample sizes. However, higher precision can be misleading, especially if studies are biased.

MA mathematically combines different study results (18); it computes summary statistics for each study, then further summarizes and interprets study-level summary statistics. Summary association estimates allow for overall judgments on investigated associations; however, the interpretation depends on assumptions and models used when combining data across studies. Observational studies are far more susceptible to confounding and bias; therefore, they have additional degrees of imprecision beyond observed CIs. Furthermore, many associations differ by study characteristics and exposure levels and types; thus, true effect size genuinely varies. Weighting studies in meta-analyses typically considers study imprecision and heterogeneity between studies, yet some also weigh quality scores (19). We generally discourage including quality scores because they are subjective, and it is difficult to summarize quality in a single number or weight. Nevertheless, when combining studies of different designs or identifying large discrepancies in risks of bias, additional subgroup or sensitivity analyses such as excluding studies with lower credibility and identifying influences of such studies in summary estimates. More sophisticated methods try to “correct” results for different types of bias related to internal validity and generalizability features in each study (20). Yet, they are not widely used and worthy of skepticism for claims to correct bias (21).

##### Fixed-Effects Model

If a single effect underlies an investigated association and all studies are homogenous, obtain a summary estimate by weighted mean by measuring that effect in fixed-effects models. The weights reflect each study’s precision. Precision is the degree of resemblance among study results if the study is repeated under similar circumstances.

Estimate precision is mainly related to variations of random error, such as sample size or the number of events of interest; measurement uncertainty—accurate and calibrated measurements—and the nature of measured phenomenon (where some events are simply more variable than others in occurrence) also affect estimate precision. The precision of estimates is expressed as the inverse variance of association estimates—or 1 divided by the square root of its standard error. Summary estimates are referred to as the fixed-effects association estimate. Fixed-effects models assume all studies have a common true (“fixed”) overall effect and any differences in observed estimates between studies are due to random error—a strong assumption typically unsuitable for most observational data.

##### Random-Effects Model

The random-effects model allows each study its own exposure or treatment association estimate with distributed associations varied across different individual and population characteristics, as well as dependent on exposure and treatment characteristics, such as dose or category. We expect sufficient statistical commonalities across studies when combining information; however, identical true association estimates are unnecessary for included studies. For example, the association between hormone therapy and the risk of cardiovascular disease among women depends on menopausal status and the type of hormone therapy. Although studies investigating hormone therapy and cardiovascular disease have exposure, population, and outcome in common, there are different true effects across different reproductive stages and formulations of hormone therapies (22).

The random-effects model is an extension of the fixed-effects model, where each study estimate combines the true total effect and difference from variation between studies and random errors. Therefore, an additional parameter represents variability between studies around the true common effect and distinguishes random-effects models from fixed-effects models. To simplify, random-effects models distribute true effect sizes represented across different studies. The combined random-effects estimates represent the mean of the population of true effects. Thus, we can generalize findings to broader phenotypes and populations beyond specific, well-defined phenotypes and populations analyzed in individual studies. For instance, an MA of observational studies on hormone therapy and cardiovascular disease provides an overall measure of association estimates between hormone therapy and cardiovascular disease; however, random-effects estimates are summary estimates of the overall true measure of association estimates of different types of hormone therapies and true measured of observed association estimates among different women’s reproductive stages. As a result, random-effects models incorporate higher degrees of heterogeneity between studies. It also gives proportionally higher weights to smaller studies and lower weights to larger studies than the fixed-effects association estimates, resulting in differences in summary estimates between the two models.

The random-effects model incorporates study variance and results to wider CIs. However, random and fixed-effects estimates would be similar, with no observed between-study variability and zero estimated between-study variance. There are many variants of random-effects models (23). Inverse variance and DerSimonian-Laird methods are the most widely used, yet these are not methods with the best statistical properties in most circumstances. Therefore, accurate working knowledge of alternatives and choosing the best-fit methods is essential (23).

We previously compared different characteristics of fixed-effects vs. random-effects in Supplementary Table S3. Since observational studies typically involve variable study populations, different levels of adjustments and analyses than RCTs, and participants under different conditions, they are usually better represented by random-effects than fixed-effects models. It is even more true when different study designs are combined or when observational studies are combined with RCTs. However, random-effects models also come with several caveats. For example, estimates of between-study variance in calculations of limited numbers of studies are very uncertain; different random-effects methods yield substantially different results; in the presence of publication selection bias (mostly affecting smaller studies), random-effects models give even more importance to smaller studies and summary estimates are more biased than fixed-effects models. Some methodologists propose methods to overcome these issues, such as only combining large enough studies, using other forms of weighting, or correcting for publication and other selective reporting biases (24–26). Familiarity with the data at hand and the suitability of methods related to specific MAs is crucial.

#### Step 3. Follow the Statistical Analysis Plan

Statistical analysis plans are designed during SR protocol preparation; we describe such plans in Step 6 of our previously published guideline (4). In addition to detailed descriptions of planned analyses, SR protocols provide descriptions of research questions, study designs, inclusion and exclusion criteria, electronic databases, and preliminary search strategies. We previously discussed review protocol preparation in detail (4). Further detailed instructions on how to prepare a statistical analysis plan can be found in Supplementary Material S3.

#### Step 4. Prepare Datasets for Meta-Analysis

Prior to MA, examine the results extracted from each study with either a dichotomous or continuous outcome (Supplementary Material S4).

If studies use different units when reporting findings, convert units for consistency before combining. Decide units (SI or conventional units) and scales (meter, centimeter, millimeter) before mathematically combining study estimates. Resolve differences in reporting summary statistics, such as measures of central tendency (mean or median) and spread (range or standard deviation). Convert studies reporting median and interquartile range (or range) to mean and standard deviation through *a priori*-defined methods, such as those described by Hozo or Wan (27, 28). Although studies not reporting summary statistics or central tendency and spread are excluded from meta-analyses, keep track of them and discuss unusable evidence and inference effects. Determine if outcomes are normally distributed. Transform values from studies reporting non-normal distributions for combination, such as log transformation.

Data reflecting risk at multiple levels of exposure, such as quantiles, present special challenges. By only extracting estimates of risk in upper versus lower levels of exposure, such as nutrient levels in nutritional associations, valuable information is lost. We suggest an interval collapsing method (29) that allows using information from all levels of exposure. Consider issues of dose-response relationships and non-linearity. Prespecify the plans for extracting and synthesizing relevant data. We advise reading and discussing articles about common MA methods on trends and dose-response (30–32). If studies use different cut-points to define exposure categories for continuous exposures, carefully record and consider them in the analysis (33).

Since most SR involves fewer than 100 studies, use simple spreadsheet applications to encode study details and association estimates. Use dedicated database management software, such as RedCap (free) or Microsoft Access (commercial). Recently popularized machine-learning-based software, such as Covidence (with limited validity), helps extract data, screen abstracts, and assess the quality and allows data transfer to RevMan (Cochrane Collaboration). RevMan is a multifunctional MA software performing qualitative and quantitative analyses and may be suitable for beginners. However, many MA methods are unavailable in RevMan, which limits analysis options. R (free) and Stata (commercial) are other softwares one may consider for data analysis (Supplementary Table S4), We also recommend mapping adjusted variables from in each study and the analyses done (main analyses and subgroup or restricted analyses). It allows a bird’s eye view of what adjustments were made, how consistent or different adjustments considered for inclusion in the MA were across different studies, and whether different unadjusted and adjusted estimates were provided in specific studies. Adjusted and unadjusted or crude association estimates across studies are often available, and differences should be accounted and explained. When preparing data analysis plans, common dilemmas include choosing among several models and the provided variably adjusted estimates. When undertaking a synthesis or review for a particular research question using causal structures, such as through DAGs, identify confounders ideally included in studies’ adjusted models in the selection criterion. When selecting estimates for MA, limit analysis to studies adjusting for confounders defined important *a priori*. Alternatively, combine different covariate-conditional estimates, such as conducting minimally adjusted and maximally adjusted analyses and comparing summary results. When combining estimates from studies with estimated different covariate-conditional effects, we advise caution regarding the non-collapsibility of odds and hazard ratios, where covariate-conditional odds ratios may differ from crude odds ratios even in the absence of confounding; however, estimates of risk ratios do not exhibit this problem (34). Ultimately, compare sensitivity analysis results between meta-analyses of adjusted and unadjusted data to indicate the presence of biases.

#### Step 5. Run the Meta-Analysis

##### Meta-Analysis for Dichotomous Outcome

The most common measures of associations for dichotomous outcomes are proportions and prevalences, risk ratios, odds ratios, relative risks, hazard ratios, or risk differences. Mathematically transform and approximately normally distribute each of these association measures into new measures on a continuous scale. Meta-analyze transformed measures using standard tools for continuous effect sizes where derived summary effects may be finally back-transformed into its original scale. We provide an overview of study designs and common transformations in Supplementary Tables S5–S7.

Originally developed as a technique for examining odds ratios with stratification in mind, the Mantel-Haenszel method was not originally developed for MA. The Mantel-Haenszel approach bypasses the need to first transform risk estimates, performs an inverse-variance weighted MA, and then back transforms summary estimators. With a weighted average of the effect sizes result, applying it directly to study risk ratios, odds ratios, or risk differences is advised. It provides robust estimates when data are sparse and produces estimates similar to the inverse variance method in other situations. Therefore, the method can be widely used. Peto’s approach is an alternative to the Mantel-Haenszel method for combining odds ratios. Peto’s summary odds ratio can be biased, especially when there are large differences in sample sizes between treatment arms; however, it generally works well in other situations. Although the Mantel-Haenszel and Peto methods pertain to raw counts with no applicability in most meta-analyses of observational data where adjusted estimates are considered, they may apply to types of observational data where raw counts are involved, such as adverse events.

When outcomes in comparison groups are either 100% or zero, computational difficulties arise in one (single-zero studies) or both (double-zero studies) comparison groups. Some studies purposely remove double-zero studies from their analyses. However, such approaches are problematic when meta-analyzing rare events, such as surgical complications and adverse medication effects. In these instances, a corrective count—typically 0.5—is added to the group with an otherwise zero count. The metan package in Stata and the metabincommand from the meta library in R correct these by default. Nyaga et al. (35) provide a guide for Stata. Such arbitrary corrections possibly introduce bias or even reverse MA results, especially when the number of samples in two groups is unbalanced (10). We advise avoiding altogether or extreme caution when using methods that ignore information from double-zero studies or use continuity corrections. Beta-binomial regression methods may be the best approach for treating such studies when computing summary estimates for relative risks, odds ratios, or risk differences (36).

##### Meta-Analysis for Continuous Outcomes

For continuous outcomes, investigate two exposure groups (exposed vs. unexposed) or per unit increase in exposure in terms of their mean outcome level. The association is quantified as the mean difference—for example, the difference between study groups in mean weight loss—or as beta-coefficient from univariable or multivariable regression models. A MA can then directly summarize mean differences for each study. If different measurement scales, such as different instruments or different formulas to derive outcomes, are available, we advise using standardized mean differences as measures of association estimates in MA—the mean difference divided by pooled standard deviation. Use one of several ways to calculate pooled standard deviation, such as the most popular methods for standardized effect sizes: Cohen’s D, Hedge’s g, and Glass’ delta (37–39).

To measure standardized size effects, combine mean, standard deviation, and sample size of exposed and non-exposed groups as input with different weights. If using software, select the standardization method. Hedge’s g includes a correction factor for small sample bias; it is preferred over Cohen’s D for very small sample sizes (fewer than 20) (39). Otherwise, the two methods give very similar results. Expressing the standardized effect measure demonstrates differences between exposed and non-exposed groups by standard deviation. For example, if Hedge’s g is 1, groups differ by 1 standard deviation and so on. When standard errors are very different between study groups, Glass’s delta—a measure using only the standard deviation of the unexposed group—is usually used to measure effect size (38). If mean differences or standardized mean differences are combined, calculate with only the effect size and standard deviation of individual groups. The software calculates the differences and associated variance of differences for weighting—the standardized mean differences with appropriate variance estimation (Supplementary Tables S6, S7, example Supplementary Figures S1, S2).

##### 95% Confidence Intervals (CIs) and Prediction Intervals

Providing 95% CIs and prediction intervals is desirable when performing a MA. CIs reflect sampling uncertainty and quantify the precision of mean summary measures of association estimates; prediction intervals reflect expected uncertainty in summary estimates when including a new study in meta-analyses. Prediction intervals—along with sampling uncertainty—reflect inherent uncertainty about specific estimates and estimate the interval of a new study if randomly selected from the same population of studies already included in meta-analyses (40, 41). Implement prediction intervals in random-effects MA frameworks. To calculate prediction intervals, 3 studies are required; however, considering prediction intervals account for the variance of summary estimates and heterogeneity, they can be imprecise for MA of few studies.

#### Step 6. Explore Heterogeneity

Cochran’s Q homogeneity test and its related metric—the Higgin’s & Thompson’s I^2^ index—are commonly used in most statistical software (Stata, R, and RevMan). Under the hypothesis of homogeneity among the effect sizes (42), the Q test follows a Chi-square distribution (with k-1 degrees of freedom, where k is number of studies). The Q test is used to evaluate the presence or absence of statistically significant heterogeneity based on α threshold of statistical significance (43). Calculated as [Q−df]/x 100, the I^2^ measures the proportion of the total variability in effect size due to between-study heterogeneity rather than sampling error. I^2^ is highly influenced by the size of the studies (within-study variability), not just the size of between-study heterogeneity. A higher percentage indicates higher heterogeneity. H is the square root of the Chi-square heterogeneity statistic divided by its degrees of freedom. It describes relative differences between observed and expected Q in the absence of heterogeneity. The H value of 1 indicates perfect homogeneity. R is the ratio of the standard error of the underlying mean from random-effects meta-analyses to standard errors of a fixed-effects meta-analytic estimate. Similar to H, the *R* ^2^ value of 1 indicates perfect homogeneity. Finally, *τ* ^2^ is the estimate of between-study variance under random-effects models. *τ* ^2^ is an absolute measure of between-study heterogeneity; in contrast to other measures (Q, I^2^, H, and R), it does not depend on study precision (44). Further information about heterogeneity can be found here (45).

##### Classification of Heterogeneity

Assessing heterogeneity in SRs is crucial in the synthesis of observational studies. Recall that the reliability of heterogeneity tests hinges on the number of studies. Thus, fewer studies make I^2^ estimates unreliable. To classify heterogeneity, different categorizations are used across different meta-analyses. The Cochrane Collaboration recommends classifying 0%–40% as likely unimportant heterogeneity; 30%–60% as likely moderate heterogeneity; 50%–90% as likely substantial heterogeneity; and 75%–100% as likely considerable heterogeneity (18). Although there is no rule of thumb for I^2^ cut-offs to classify studies as low, medium, or high heterogeneity, categorize using *a priori* protocol definitions. Provide CIs for I^2^ since estimates of heterogeneity have large uncertainty (46) (See Supplementary Figures S1, S2 for examples).

##### Subgroup or Restricted Analysis

Ideally, all studies compared in meta-analyses should be similar; however, it is almost impossible for observational studies. When performing subgroup analyses, look at factors explaining between-study heterogeneity. Explore subgroups, including patient or individual characteristics, study methods, and exposure or outcome definitions. Define subgroup characteristics *a priori*. Group studies according to study characteristics. We outline a subgroup analysis essential guide in Supplementary Table S8 (Supplementary Figure S3 provides example).

##### Meta-Regression

Meta-regression applies basic regression concepts using study-level association estimates (42, 47, 48). Examining the association—typically linear, yet not in all cases—between the outcome of interest and covariates determines the contribution of covariates (study characteristics) in the heterogeneity of the association estimates. In common regression analyses, patient-level information is used when comparing outcomes and exposures alongside various covariates. In meta-regression (instead of patient-level information) use population-level information, such as mean age, location, mean body mass index, percentage of females, mean follow-up time, and risk of bias, to explore association estimates. The common practice of visualizing meta-regressions is with bubble plots (Supplementary Figure S4) using the metareg package in Stata (49).

In meta-regression, variables under investigation are potential effect modifiers. Beta-coefficient refers to incremental changes in outcomes with increasing levels of the covariate. Positive coefficients signify an increase in the outcome with increasing levels of the covariate variable; negative coefficients mean a decrease in the outcome.

It is important to understand that meta-regression explores consistency of findings and does not make causal inferences on associations. Meta-regression results are based on observational data across different studies. Thus, it suffers from similar pitfalls in causality and biases. A statistically significant association between an outcome and covariate (beta coefficient) may have a confounding variable that drives the association, albeit occasionally mitigated by multivariate analysis. In addition, covariates, in some cases, can be highly collinear. Since most SR involve fewer studies capable of meta-regression, power is also an issue. The number of studies is one major stumbling block when performing meta-regression. In multivariable analysis, the number of studies becomes more important since more studies are required. Based on recommendations from the Cochrane Handbook for Systematic Reviews of Diagnostic Test Accuracy, do not consider meta-regression with fewer than 10 studies in a MA. For multivariable regression, they advise at least 10 studies per covariate (50), which means multivariable analysis requires at least 20 studies (47). Meta-regression may also be subject to ecological fallacy. In meta-regression, we use average study participant characteristics; therefore, the association between average study participant characteristics and measures of association estimate may not be the same within and between analyzed studies. Common covariates prone to ecological fallacy are age and sex. Using individual-level data is the only way to avoid ecological fallacies (51). Use caution if concluding causality from meta-regression and interpreting results (52). False positive claims are common in meta-regression (50).

While the most commonly used meta-regression is the random-effects meta-regression, other models, such as fixed-effects meta-regression, control rate meta-regression, multivariate meta-regression and Bayesian hierarchical modeling, can be used. These methods will depend on the specifics of analysis, such as the type of data, the number of studies, and the research question. More information can be found elsewhere (53, 54).

#### Perform Leave-One-Out Analysis (Influence Analysis)

An MA may include studies providing extreme positive or negative associations. Sometimes it is possible to identify such outliers visually by expecting the forest plot, but often the situation is more complex due to sampling variances across included studies (55). To explore whether the outlier influences the summary effect estimate, one can explore whether the exclusion of such study from the analysis leads to considerable changes in the summary effect estimate. In case of small number of studies, the exclusion may be done manually; yet the most commonly used statistical software provide a possibility to perform a leave-one-out analysis, which iteratively removes one study at a time from the analysis and provides recomputed summary association estimates (48). For instance, in STATA, use the metaninf package (56) or in R, use the metafor package to perform a leave-one-out analysis (example shown in Supplementary Figure S5). For further reading, we suggest the article on outlier and influence diagnostics for MA (55).

#### Step 7. Explore Publication Selection Bias

Selection bias related to the publication process—or publication selection bias—arises when disseminating study results influences the nature and direction of results (57). Publication selection biases include: a) classic publication bias or file drawer bias when entire studies remain unpublished; time-lag bias when rapid publication depends on results; b) duplicate publication bias when some data are published more than once; c) location bias or citation bias when citations and study visibility depend on results; d) language bias when study publication in different languages is differentially driven by results; and e) outcome reporting bias when only some outcomes and/or analyses are published preferentially.

A thorough literature search is the first step in preventing publication bias (explained in our previous publication) (4). In addition to bibliographic database search, rigorous search of the gray literature and study registries (for preliminary data or for unpublished results) should be done to identify other studies of interest. We summarized the most important databases in Supplementary Table S9. In addition, one should consider whether highly specialized or very large numbers of studies without any special planning (e.g., when exposures and outcomes are commonly and routinely measured in datasets such as ubiquitous electronic health records) readily address the question of interest. Selective reporting bias is very easy to be introduced in the latter situation.

Several methods exist for exploring publication selection bias; however, no method definitively proves or disproves publication selection bias. We comment on several widely popular, yet often over-interpreted methods in the next two subsections and in Supplementary Table S10 and we urge caution against their misuse and misinterpretation. Based on statistical properties (sensitivity and specificity for detecting publication selection bias), newer tests, such as those based on evaluating excess statistical significance (26), may perform better. When less biased summary estimates of effects are desired, the Weighted Average of Adequately Powered Studies (WAAP) (24) (that focuses on studies with >80% power) may have the best performance. However, many MA has few studies and not well-powered studies at all; then any test for publication selection bias and attempt to adjust for such bias may be in vain. Even greater caution is needed in such circumstances.

#### Visual Inspection of Study Results

To help understand whether effect sizes differ systematically between small and large studies, funnel plots provide the simplest technique and a graphical representation (Supplementary Figure S5). Funnel plot graphs demonstrate association sizes or estimates on the horizontal axis (x-axis) and the study precision, sample size, or the inverse of the standard error on the vertical axis (y-axis)—an inverted funnel. Ideally, symmetry around the estimates provided by larger studies (the tip of the inverted funnel) extends to the smaller studies (the foot of the inverted funnel). An asymmetrical funnel shape with larger estimates for smaller rather than larger studies hints at publication selection bias, yet other possible reasons exist for the same pattern. Draw cautious inferences (58, 59). Since plain visual assessment is subjective, we do not recommend using it as the sole criterion to arbitrate publication bias.

In some observational studies, observed differences between large and small studies arise from methodological differences. Different study characteristics in study sizes can lead to heterogeneity in the analysis. For example, smaller studies can have more stringent disease criteria for inclusion (lower risk for misclassification bias) and more intricate methods for data collection (lower risk for recall bias) compared with larger studies. More commonly, smaller studies are subject to more selective analysis and reporting pressure with possibly more bias than well-designed large studies. There is no way to generalize *a priori* for all topics, and studies should be examined carefully in each case. Thus, in the context of observational studies, it holds even more than funnel plot asymmetry should not automatically indicate publication bias (9, 10). In particular, any factor associated with both study effect and study size could confound the true association and cause an asymmetrical funnel. Contour-enhanced funnel plots may help interpret funnels and differentiate funnel plot asymmetry caused by statistical significance-related publication bias from other factors; however, most of these caveats still apply (60).

#### Statistical Tests to Explore Publication Selection Bias

Several tests and statistical methods are developed to detect (and potentially correct) publication selection bias. Egger’s test remains the most popular. It is based on linear regression of normalized association or effect estimates (using association estimates divided by standard errors) and study precision (inverse of the standard error) (61, 62). The intercept of regression lines measures the asymmetry—the larger its deviation from zero, the bigger the funnel plot asymmetry. A *p-value <0.05* indicates the presence of publication bias, which means estimates of smaller studies do not mimic estimates of larger studies. Egger’s test may be unreliable for fewer than 10 studies. We advise caution when interpreting estimates of fewer than 10 studies. Further, for log odds ratios, even in the absence of selective outcome reporting, the test inflates Type I errors (false positive findings) (58, 63). When all studies have similar variances, test results have no meaning. Egger’s test (and other modifications) as small study effect tests (i.e., whether small and larger studies give different results) should be used rather than strictly as a test of publication selection bias (See Supplementary Figures S6, S7 for example).

Other methods have been developed to address the limitations of existing popular approaches, such as the three-parameter selection model (64), the proportion of statistical significance test (26), and variants thereof. The three-parameter selection model’s main assumption is the likelihood of publication is an increasing step function of the complement of a study’s *p-value*. Maximum likelihood methods estimate corrected effect sizes and the relative probability that insignificant results are published. Whereas the proportion of statistical significance test compares expected with observed proportions of statistically significant findings. Find detailed explanation elsewhere (26). Some methodologists propose the most reliable summary results are obtained by methods accommodating possibilities of publication selection bias. With proven, good statistical properties, some of these methods may be used more in the future (26). However, for typical meta-analyses with limited available data, mostly small studies, and no formal pre-registration, no methods are likely perfect. Even when not formally demonstrated, consider publication selection bias as a definite possibility.

## Discussion

Synthesizing data from high-quality observational studies, at low risk of bias, complements data from RCTs and may provide insight into prevalence, the generalizability of findings for different populations, and information on long-term effects and desirable or adverse events (harms) when dealing with interventions. SRs and MA help quantify associations not testable in RCTs, such as quantifying the association between age at menopause onset or obesity with health outcomes. For observational evidence which assess interventions, we recommend applying the grading of recommendations, assessment, development, and evaluation (GRADE) tool to translate results from SRs and MA into evidence-based recommendations for research and clinical and public health impact (65). Applying GRADE addresses a range of research questions related to diagnosing, screening, preventing, treating, and public health. A panel of experts formulates recommendations, ideally experienced information specialists and subject matter experts. For observational evidence pertaining to putative protective and risk factors, use a series of criteria focused on the amount of evidence, statistical support, the extent of heterogeneity, and hints of bias (66). Eventually, systematic reviews and meta-analyses are observational studies themselves. Therefore, always cautiously interpret and take special care when claiming causality and framing strong recommendations for policy and clinical decision-making.
