# Supplementary material for: A 7-Step Guideline for Qualitative Synthesis and Meta-Analysis of Observational Studies in Health Sciences
Source: Public Health Rev. 2023 May 16;44:1605454. doi: 10.3389/phrs.2023.1605454 (PMC10227668; doi:10.3389/phrs.2023.1605454)
Supplement: Supplementary file 1 [file DataSheet1.docx]

**A 7-step guideline and checklist for qualitative synthesis and meta-analysis of observational studies in health sciences**

**Supplementary Materials**

**Authors**

Marija **Glisic***^1,2^, Peter Francis **Raguindin***^1,2,3,4^, Armin **Gemperli**^2,5^, Petek Eylul **Taneri**^6^, Dante Jr **Salvador**^1,3^, Trudy **Voortman**^7,8^, Pedro **Marques Vidal**^9^, Stefania I **Papatheodorou**^10^, Setor K **Kunutsor** ^11,12^, Arjola **Bano**^1,13^, John P.A. **Ioannidis**^14,15,16,17^ Taulant **Muka**^1, 17, 18^

*Denotes equal contribution/ Shared first authorship

**Author affiliation**

^1^ Institute of Social and Preventive Medicine, University of Bern, Bern, Switzerland

^2^ Swiss Paraplegic Research, Nottwil, Switzerland

^3^ Graduate School for Health Sciences, University of Bern, Bern, Switzerland

^4^ Faculty of Health Science and Medicine, University of Lucerne, Lucerne, Switzerland

^5^ Institute of Primary and Community Care, University of Lucerne, Lucerne, Switzerland

^6^ HRB-Trials Methodology Research Network, National University of Ireland, Galway, Ireland

^7^ Department of Epidemiology, Erasmus MC, University Medical Center, Rotterdam, Netherlands

^8^ Division of Human Nutrition and Health, Wageningen University & Research, the Netherlands

^9^ Department of Medicine, Internal Medicine, Lausanne University Hospital (CHUV) and University of Lausanne, Lausanne, Switzerland

^10^ Department of Epidemiology, Harvard TH Chan School of Public Health, Boston, Massachusetts, US

^11^ Diabetes Research Centre, University of Leicester, Leicester General Hospital, Gwendolen Road, Leicester, LE5 4WP, United Kingdom

^12^ Translational Health Sciences, Bristol Medical School, University of Bristol, Learning & Research Building (Level 1), Southmead Hospital, Bristol, United Kingdom

^13^ Department of Cardiology, Inselspital, Bern University Hospital, University of Bern, Bern, Switzerland

^14^Stanford Prevention Research Center, Department of Medicine, Stanford University School of Medicine, Stanford, CA, United States,

^15^Department of Epidemiology and Population Health, Stanford University School of Medicine, Stanford, CA, United States,

^16^Department of Statistics, Stanford University, Stanford, CA, United States,

^17^Meta-Research Innovation Center at Stanford (METRICS), Stanford University, Stanford, CA, United States, ^18^Epistudia, Bern, Switzerland

**Corresponding Author**

Taulant Muka, MD, MPH, PhD

Epistudia

Bern, Switzerland

[taulant.muka@epistudia.ch](mailto:taulant.muka@epistudia.ch)

**Table of Contents**

[**Supplementary Material I.** The main characteristics of observational study design 3](#_Toc131577606)

[**Supplementary Material II.** Quality assessment 4](#_Toc131577607)

[**Supplementary Material III.** Preparing statistical analysis plan 5](#_Toc131577608)

[**Supplementary Material IV.** Data extraction form 6](#_Toc131577609)

[**Table S1.** Information needed to meta-analyze specific association estimates 7](#_Toc131577610)

[**Table S2.** Checklists and scales used for quality assessment of individual studies 8](#_Toc131577611)

[**Table S3**. Statistical model for meta-analysis 9](#_Toc131577612)

[**Table S4.** Statistical software for meta-analysis 10](#_Toc131577613)

[**Table S5**. Observational Study Design Characteristics 11](#_Toc131577614)

[**Table S6.** Overview on effect sized measures for dichotomous data, with normalizing transformation and example codes in Stata (metan) and R (meta/metacont) 12](#_Toc131577615)

[**Table S7**. Meta-analysis for continuous outcomes using Stata (metan) and R (meta/metacont) 14](#_Toc131577616)

[**Table S8.** Subgroup analyses guidelines 15](#_Toc131577617)

[**Table S9.** Possible database for searching grey literature 16](#_Toc131577618)

[**Table S10.** Tests for publication selection bias 17](#_Toc131577619)

[**Figure S1.** Sample output on the Stata metan command. 18](#_Toc131577620)

[**Figure S2.** Forest plot on the weighted mean difference of triglyceride levels between individuals with tetraplegia and paraplegia. 19](#_Toc131577621)

[**Figure S3.** Forest plot on the weighted mean difference of triglyceride levels between individuals with tetraplegia and paraplegia grouped according to age. 20](#_Toc131577622)

[**Figure S5.** Graph and test readout from Stata metaninf package on leave-one out analysis using the comparison of triglyceride levels between tetraplegia and paraplegia. 22](#_Toc131577623)

[**Figure S6.** Funnel plot of studies comparing the triglyceride levels of tetraplegia and paraplegia. 24](#_Toc131577624)

[**Figure S7.** Read-out of the test for small study effects (Egger’s regression) from Stata using metabias. 25](#_Toc131577625)

[**References** 26](#_Toc131577626)

# **Supplementary Material I.** The main characteristics of observational study design

A study can be prospective, where person-time begins after the study started and exposure and outcome information are collected prospectively, or retrospective where person-time begins before and after participants have already developed the outcomes of interest. In a cohort study, participants are identified, based on a target population, exposures are measured, and individuals are followed up over time where an outcome is ascertained. In a case-control study, those who have an outcome of interest, the cases, are selected and their exposures are compared with those without the outcome, the controls. Controls are selected that represent the source population where the cases were chosen from, where the confounder and exposure distribution among controls should be the same as it is in the source population of cases. There are several methods for control selection, including cumulative sampling, where controls are selected among the non-cases at the end of follow-up, case-cohort sampling, where controls are from those who were at risk of the outcomes at the beginning of follow-up, and incidence density sampling, where controls are selected from the non-cases at the time each case occurs as they are followed through time, among others. A case-control study may also be nested within a cohort study or can be conducted outside of an established cohort study. Special attention to how controls are selected is warranted as this can influence biases, as has been demonstrated by the potential for selection bias and bias from missing data from hospital-based controls (Berkson bias).(1) A cross-sectional study includes all persons, or a representative sample, in a population at one point in time who are selected without regard to exposure or disease status and are not followed through time. Particularly, concerning in cross-sectional studies is the lack of temporality between an exposure and an outcome, a property for establishing causality. An ecological study is conducted on a group or cluster of people and where data are aggregated and do not pertain to an individual. Often in ecological studies, it is difficult to appropriately control for confounding, exposure data is often only available at the area level which may lead to misclassification, and the potential for the ecological fallacy, where associations observed at the aggregate level do not reflect those at the individual level.(2) There are also hybrid study designs which can be used often to address a limitation of different designs, for example combining ecological data from administrative registries and cohort individual-level data to address missing ecological confounders.(3) A special case of observational study is a post-trial observational follow-up study. The studies, although valuable tool to increase our understanding of long-term effects of interventions, these post-trial observational studies are cohort studies susceptible to bias and their findings should be interpreted with caution.

Studies may report an effect estimate or an estimate of association, confidence intervals, p-values, standard errors derived from a statistical model, depending on the study design, to describe the relationship between an exposure and an outcome (**Table S5**). Estimates of effects derived in observational studies include those of prevalence, the proportion of a population in a state of time and incidence, the measurement of new events or changes in health states or outcomes. Specific measures include, prevalence measures of prevalence difference, prevalence ratio, prevalence odds ratio, and incidence measures of risk difference, risk ratio, incidence odds ratio, incidence rate ratio, and incidence rate difference for dichotomous outcomes, whereas the difference of means are reported for continuous outcomes. As an example, logistic regression models may be used to estimate an odds ratio (the most common effect estimate) where the parameter of interest for a meta-analysis, β, denotes the covariate-adjusted natural log of the odds ratio per unit of exposure, where the exposure represents a unit change for either a binary, ordinal, or continuous variable. Estimates in studies should be reported as both unadjusted and adjusted for a set of potential confounders, where there may be several different adjusted models reported with a different set of confounders; however, many studies may fail to report unadjusted estimates.

# **Supplementary Material II.** Quality assessment

Similar to screening and selecting articles, the quality of individual studies should be assessed by at least two independent reviewers, who should reach an agreement in cases where overall assessments of individual studies are different. If there is a disagreement between two reviewers on quality assessment, a third, more experienced reviewer should help reach a consensus. Various tools, including checklists and scales, have been developed to assess the quality of individual observational studies. Checklists are used to assess reporting and methodological quality, while scales are used to assess methodological quality. These tools typically consist of questions assessing if potential biases have been considered. These domains include participant selection, exposure and its assessment, outcome and its assessment, confounding, comparability of study groups, and statistical analyses. Individual studies are then assessed against the checklist, whether these considerations were reported or not. Examples are The Agency for Healthcare Research and Quality (AHRQ), Critical Appraisal Skills Program (CASP) checklist, Joanna Briggs Institute (JBI) checklists, National Institutes of Health (NIH) quality assessment tool (4, 5, 6, 7), **Table S2**. Scales make graded assessments of studies to assess methodological quality. The most commonly used scale is Newcastle Ottawa Scale (NOS) (8). NOS was developed for case-control and cohort studies but has also been adapted for cross-sectional studies (9, 10). Individual studies are assessed based on three domains: participant selection, comparability of study groups, and ascertainment of exposure (case-control) or outcome (cohort and cross sectional) of interest (8). Studies are graded with a maximum of 9 stars (low risk of bias), 7-8 stars (moderate risk of bias), and 6 or less stars (high risk of bias)(8)^,^(11). While checklists may provide information on the quality of reporting of items (present vs absent reporting), scales may give a more comparable assessment because of explicit grading. The use of scales aids the reviewers in providing a more detailed, structured assessment of studies and can help minimize, albeit not fully eliminate, the subjectivity of assessment. Moreover, subgroup analyses and meta-regression analyses may also utilize methodological assessment grades in the exploration and explanation of the heterogeneity of results. For example, studies may be synthesized and stratified by risk of bias. The information needed to assess studies using the scales will have been considered in the checklists. Hence graded assessments can be performed simultaneously with the checklist assessments. Scales may also facilitate the development and synthesis of evidence and recommendations using GRADE. As such, studies with a low risk of bias are prioritized over those with a high risk of bias. In case the meta-analysis includes studies of poor quality, we can evaluate whether the quality of studies influences the pooled results by performing a sensitivity analysis that excludes studies of poor quality. The choice of an appropriate design-specific quality assessment tool should be guided by the research question, and not by the design of the individual studies. We provide two examples: (i) Included studies may have a prospective design relative to study objectives but may have a cross-sectional design relative to the review objectives. (ii) Included studies may have a case-control design (i.e., a disease state is an outcome in the design), but the outcome of the individual studies may correspond to the exposure in the systematic review research question. In these cases, the selection of the quality assessment tool should be based on the review question.

# **Supplementary Material III.** Preparing statistical analysis plan

When preparing statistical analysis plans, consider how the plan answers the following questions:

1. What is the direction and size of the summary association estimate?
2. What is the statistical heterogeneity?
3. Does a single study drive the overall association estimate?
4. What study and study population characteristics influence the size of pooled association estimate?
5. Are the results reliable?
6. Is there a serious risk of reporting bias, such as publication or selective bias?

Prepared *a priori*, meta-analyses rigorously follow data analysis plans. Specify any additional or unplanned analyses *a posteriori* analyses, then amend study protocols and report additional analyses. For example, high methodological heterogeneity among studies restricts analysis to subgroups after data extraction. Thus, thorough and complete data extraction is imperative. We provide general advice on organizing data extraction in **Supplementary Material IV**.

To plan statistical analyses, one should understand study estimates across observational studies as a prerequisite. We provide brief descriptions of observational study characteristics and properties worthy of consideration when conducting evidence synthesis and meta-analysis (**Supplementary Material I** and **Table S3**). For further details, we advise consulting other resources (12, 13). To combine overall association estimates, we overview requisite information extracted from original studies in **Box 1**. In cases where sufficiently large numbers of studies are available for meta-analysis, we recommend specific extracted data needed for subgroup or sensitivity analyses, such as methodological features, risk of bias of available studies, and study population characteristics—we discuss details in **Section 7.1.**

# **Supplementary Material IV.** Data extraction

At the beginning of the data extraction, each selected study must be evaluated for eligibility according to the study protocol and checklist; only the studies that fulfill inclusion criteria should be included in the data extraction form. Data extraction forms should be prepared carefully to define all the included studies properly and help for the further steps in the qualitative and quantitative synthesis. The information you will extract should be based on *a priori* decision, and it will depend on your research questions and subgroup analysis you will plan to conduct, as well as potential factors you will be exploring as the source of heterogeneity. In general, it is important to extract information related to author name, publication year, title of the study, study design, location, study (cohort’s) name, duration (follow-up time), number of participants, % of the female participants, number of the events, age mean and standard deviation of the participants, obesity, ethnicity, definition/assessment of the exposure and outcome, levels of adjustments, analysis type, estimates and their 95% confidence intervals or standard error for each adjustment level, funding(private vs. public) and risk of bias/quality assessment. The extraction must be brief with clear abbreviations, consistent definitions and same units, and data to be if necessary (14). It is recommended to conduct extraction with at least two independent researchers to minimize errors and potential biases. If the form was created by another researcher, the ones who would do the extraction should receive training and orientation ahead of the procedure(15).

If there are multiple published articles that are based on the data from the same study, in general, the data from the most recent publication and/or with the most complete information (e.g., larger sample size) must be included to the extraction form. Data extractors must carefully read the full text and avoid replacing it with text searching since the terminology can vary between different studies, which may lead to missing data in the form(15).

Extraction can be done with paper forms, electronic forms, and data systems. Paper forms could be insufficient and cause errors since they need to be entered into a database or a spreadsheet, compiled, and processed for analysis. Electronic forms might be more efficient in collecting data and result in fewer errors than paper forms (Adobe Acrobat, Microsoft Word, Microsoft Access, Microsoft Excel, Google Forms). Data systems (EPPI-Reviewer, Systematic Review Data Repository (SRDR), DistillerSR (Evidence Partners), Doctor Evidence) can be a more sophisticated alternative since they can be integrated with title/abstract, full-text screening and export data directly to analysis software but that would require investment to set up these commercial systems and also train data extractors (15, 16). There are automatic data extraction tools; however, the usage of manual methods is still more common (15).

# **Table S1.** Information needed to meta-analyze specific association estimates

|  | |
| --- | --- |
| **Association estimates** | **Extracted information from original studies** |
| **Prevalence** | Study name (ID)  Study size  Number of cases  Prevalence  95% confidence interval |
| **Odds ratio**  **Hazards ratio**  **Relative risk** | Study name (ID)  Number of cases  Number of events in cases  Number of non-events in cases  Number of controls  Number of events in controls  Number of non-events in controls |
| **Correlation coefficient** | Study name (ID)  Study size  Correlation coefficient  95% confidence interval |
| **Beta from regression coefficient** | Study name (ID)  Study size  Beta coefficient  95% confidence interval |
| **Mean difference** | Study name (ID)  Number of individuals in group 1  Mean value in group 1  Standard deviation of the mean in group 1  Number of individuals in group 2  Mean value in group 2  Standard deviation of the mean in group 2 |

# **Table S2.** Checklists and scales used for quality assessment of individual studies

|  | Checklists | | | | Scales | |
| --- | --- | --- | --- | --- | --- | --- |
|  | JBI | CASP | NIH | AHRQ | NOS | ROBINS-I |
| Observational Cohort | 🗸 | 🗸 | 🗸 |  | 🗸 | 🗸 |
| Case control | 🗸 | 🗸 | 🗸 |  | 🗸 | 🗸 |
| Cross sectional | 🗸 |  | 🗸 | 🗸 | * | 🗸 |

******* *Adapted versions*

***Abbreviations:*** *AHRQ – Agency for Health Research and Quality;* *CASP – Critical Appraisal Skills Programme; JBI – Joanna Briggs Institute; NIH – National Institutes of Health; NOS – Newcastle Ottawa Scale; ROBINS-I – Risk Of Bias In Non-Randomized Studies of Interventions*

| **Table S3**. Statistical model for meta-analysis | | |
| --- | --- | --- |
|  | Fixed effect model | Random effects model |
| Assumption | All studies share a common true overall effect.  The differences in observed effect between studies is due to random noise. | Each study has an effect of its own, where each study effect being the combination of a true overall effect, plus a difference coming from between-study variation and random noise. |
| True effect | One true effect. | True effect varies. |
| Interpretation of pooled estimate | Pooled estimate is common effect. | Pooled effect is mean effect of all true study effects. |
| Goal of study | Goal is to estimate the true effect for the identified population. | Goal is to estimate the mean of all true effect sizes. |
| Variability accounted for | Accounts for within study variability. Between-study variability is ignored. | Accounts for within study and between study variability. |
| When to pool the effects? | All study characteristics are identical to a large degree. | Studies have enough in common to statistically pool the information, but it is not required that these studies have an identical true effect size. |
| Interpretation of target population | Inference is relevant for the (homogenous) population included in the study. | Inference is relevant for the (heterogenous) population types included in the study. |
| Implication | Large studies are more influential (receive more weight) in analysis as compared to random effects model. | Large studies are less influential (receive less weight) in analysis as compared to fixed effect model: a more equal distribution of weights. |
| Under heterogeneity | More likely to lead to incorrect statistically significant findings than in the random effects model. | Larger p-values and wider confidence intervals than in the fixed effect model. |
| Without heterogeneity | Correct statistical inference. | Correct mean estimate, with too wide confidence intervals and too large a p-value. |
| Models for continuous outcomes | Inverse-variance fixed effect | Inverse-variance random effects  (DerSimonian-Laird) |
| Models for binary outcomes |  |  |
| - - Risk ratio | Inverse-variance fixed effect;  Mantel-Haenszel | Inverse-variance random effects  (DerSimonian-Laird) |
| - - Odds ratio | Inverse-variance fixed effect;  Mantel-Haenszel;  Peto | Inverse-variance random effects  (DerSimonian-Laird) |
| - - Risk difference | Inverse-variance;  Mantel-Haenszel | Inverse-variance random effects  (DerSimonian-Laird) |

# **Table S4.** Statistical software for meta-analysis

| **RevMan**  The Cochrane Collaboration produces RevMan software for meta-analyses. RevMan’s interface integrates data extraction, quality assessment, and summary tables. It also performs basic meta-analysis statistics (pooling estimates, heterogeneity analyses, and forest plots). However, small study effects and leave-one-out analyses are not possible with RevMan and meta-regression functions are limited. Read <https://training.cochrane.org/handbook/current> for detailed instructions and tutorials.    **Stata®**  Stata is a paid statistical program that performs meta-analysis. The free metan package contains the basic tools for pooling association estimates for continuous and dichotomous variables. Add-on packages include metareg (for meta-regression), metabias (for small study effects), and metaninf (for leave-one-out analysis). The latest version of Stata contains a meta-analysis suite with the add-on packages. Each package’s help function provides detailed instructions. Newer versions of Stata (version 16 and above) include a meta-analysis feature. Read about new feature details on the distributor’s website.  **R**  R is a free programming tool with a broad range of statistical packages for performing meta-analysis. The meta, metacont, and dmetar package is freely available and downloaded from GitHub. The package contains tools necessary for pooling continuous and dichotomous outcomes. It also contains meta-regression, small-study effect, leave-one-out analysis (influence analysis). For detailed instructions, read  <https://bookdown.org/MathiasHarrer/Doing_Meta_Analysis_in_R/heterogeneity.html>) |
| --- |

# **Table S5**. Observational Study Design Characteristics

| **Study Design** | **Sampling** | **Unit of Observation** | **Time period** | **Exposure/**  **Outcome** | **Estimates of Effect/ Association** | **Considerations for meta-analysis** |
| --- | --- | --- | --- | --- | --- | --- |
| Cohort Study | Sampled from those free of disease  /outcome at baseline | Individual | Longitudinal | Exposure and outcome are measured separately, longitudinally | -Risk Difference  -Risk Ratio  -Incidence Odds Ratio  - Incidence Rate Ratio  - Incidence Rate Difference  -Hazard Ratio  -Mean Differences | -temporality may be established  -loss to follow up may be more present  -selection bias |
| Case Control | Sampled based on disease/  outcomes | Individual | Longitudinal | Outcomes are selected first and exposure assessment occurs prior (retrospectively) | -Odds Ratio | -potential recall bias of exposures  -potential selection bias of controls |
| Cross-sectional | Sampled without regard to exposure and/or outcome | Individual | At one point in time, without follow up | Exposure and outcome are measured at the same time | -Prevalence Difference  Prevalence Ratio  -Prevalence Odds Ratio | -difficult to assess temporality  -may be prone to reverse causality. -good for prevalence-based questions |
| Ecological | Sampled with regard to the group, either based on  the method of exposure measurement  and the method of grouping | A group of people | Longitudinal | Assessed as an aggregate within the population | - Correlation coefficient  -Beta from regression coefficient  -Rate Ratio | -prone to ecological fallacy: associations observed at the aggregate level may not hold at the individual level  -difficult to measure all confounders |

# **Table S6.** Overview on effect sized measures for dichotomous data, with normalizing transformation and example codes in Stata (metan) and R (meta/metacont)

|  | | Risk ratio | Odds ratio | Risk difference |
| --- | --- | --- | --- | --- |
| Effect size  Where:  P_treat_, P_contr_: Probability of event in treatment and control arm  n1, n2: number of events in treatment and control arm  N1, N2: sample size in treatment and control arm  For the odds ratio notation we use: n1=A; N1-n1=B; n2=C; N2-n2=D | | Ratio of the probability of an event of both arms:  ES=P_treat_/P_contr_  =(n1/N1) / (n2/N2) | Ratio of the odds of the event in one arm to the odds of the event in the other arm:  ES= Odds_treat_/Odds_contr_  = A*D / B*C | Difference in risks of an event of both arms:  ES=P_treat_ – P_contr_  =(n1/N1) – (n2/N2) |
| Interpretation | | Risk ratio of 1 means that the risk is the same in both arms, indicating no effect, while a risk ratio less or greater than 1 would mean that the risk was relatively lower or higher in one or the other arm, respectively | Odds ratio of 1 means that the odds is the same in both arms, indicating no effect, while a odds ratio less or greater than 1 would mean that the odds was relatively lower or higher in one or the other arm, respectively | Risk difference of 0 means that the risk is the same in both arms, indicating no effect, while a negative of positive risk difference would mean that the risk was relatively lower or higher in one or the other arm, respectively |
| Usage | | Used in prospective studies (cohort studies). Cannot be used in retrospective (case control) studies | Commonly used as output of logistic regression analyses | Less common than risk ratio or odds ratio, but better understandable |
| Transformation | | TES=log(ES) | TES=log(ES) | TES=ES  (no transformation needed) |
| Variance of transformed value (resp. standard error squared) | | SE_TES^2^=1/n1-1/N1+1/n2-1/N2 | SE_TES^2^=1/A+1/B+1/C+1/D | SE_TES^2^=(n1/N1)*(1-(n1/N1))/N1 +  (n2/N2)*(1-(n2/N2))/N2 |
| Stata | Direct computation of inverse-variance estimation in Stata, fixed effect model | metan A B C D, fixedi rr | metan A B C D, fixedi or | metan A B C D, fixedi rd |
|  | Computation of inverse-variance estimation via transformed effect sizes, fixed effect model | metan TES SE_TES, eform | metan TES SE_TES, eform | metan TES SE_TES |
|  | Option to use random effects instead of fixed effect model | ⟵ ~~fixedi~~ randomi ⟶ | | |
|  | Option for Mantel-Haenszel (fixed effect model) | ⟵ ~~fixedi~~ fixed (default option) ⟶ | | |
|  | Option to use Peto’s method |  | ~~fixedi~~ peto |  |
| R | Direct computation of inverse-variance estimation, fixed effect model | metabin(n1, N1, n2, N2, data=dat, method="Inverse", comb.fixed=TRUE, comb.random=FALSE, sm="RR") | metabin(n1, N1, n2, N2, data=dat, method="Inverse", comb.fixed=TRUE, comb.random=FALSE, sm="OR") | metabin(n1, N1, n2, N2, data=dat, method="Inverse",  comb.fixed=TRUE, comb.random=FALSE, sm="RD") |
|  | Computation of inverse-variance estimation via transformed effect sizes, fixed effect model | metagen(TES, SE_TES, comb.fixed=TRUE, comb.random=FALSE, sm="RR", data=dat) | metagen(TES, SE_TES, comb.fixed=TRUE, comb.random=FALSE, sm="OR", data=dat) | metagen(TES, SE_TES, comb.fixed=TRUE, comb.random=FALSE, sm="RD", data=dat) |
|  | Option to use random effects instead of fixed effect model | ⟵ comb.fixed=FALSE, comb.random=TRUE ⟶ | | |
|  | Option for Mantel-Haenszel (fixed effect model) | ⟵ method="MH" ⟶ | | |
|  | Option to use Peto’s method |  | method="Peto" |  |

# **Table S7**. Meta-analysis for continuous outcomes using Stata (metan) and R (meta/metacont)

|  | Stata | R |
| --- | --- | --- |
| Data input (for each study) | Size of group 1 and 2: n1, n2  Mean effect size in group 1 and 2: m1, m2  Standard deviation of effect in group 1 and 2: sd1, sd2 | |
| Method | Inverse variance weighting | |
| Assumptions | Normality in effect sizes within trial arms.  Between trial variations in standard deviations are attributed to differences in precision, and are assumed equal in both study arms. | |
| Fixed effect estimate | metan n1 mean1 sd1 n2 mean2 sd2, nostandard fixed | metacont (n1, m1, sd1, n2, m2, sd2,  comb.fixed=TRUE, comb.random=FALSE,  sm="MD", data=dat) |
| Random effects estimate | metan n1 mean1 sd1 n2 mean2 sd2, nostandard random | Metacont (n1, m1, sd1, n2, m2, sd2,  comb.fixed=FALSE, comb.random=TRUE,  sm="MD", data=dat) |
| Random effects estimate, standardized mean differences (Hedges g) | metan n1 mean1 sd1 n2 mean2 sd2, hedges random | metacont(n1, m1, sd1, n2, m2, sd2,  comb.fixed=FALSE, comb.random=TRUE,  sm="SMD", method.smd="Hedges",  data=dat) |

# **Table S8.** Subgroup analyses guidelines

|  |
| --- |
| **Pre-specify study characteristics**.  Before conducting the review, prespecify study characteristics as part of data analysis plans. Outlining important characteristics beforehand avoids factors without scientific basis, limits the number of analyses, prevents undue influence from identified studies in the analysis, and avoids concluding from statistically random results.  Identifying relevant study characteristics beforehand is extremely challenging since the literature content is unknown. With subgroup analyses, considering study quality and risk of bias during the review process are crucial factors. For other study characteristics, consult subject matter experts. Justify exploring study characteristics deemed extremely important in clinical settings yet overlooked during the planning phase.  **Check scientific plausibility for desired subgroup analyses.**  If statistically or biologically plausible, explore specific variables in subgroup analyses. When classifying variables, clearly determine whether desired subgroup variables are effect modifiers, confounders in relationships of exposure and outcome, or different exposure and outcome definitions affecting overall results.  When investigated in subgroup or restricted analyses, effect modifiers are ideal variables since they determine association estimate differences according to subgroup variables. Since meta-regressions adjust for known variables, it is suitable for studying subgroup analyses, as well as confounders and predictor variables.  **Limit subgroup analyses.**  Since multiple subgroup analyses can lead to falsely significant findings from the multiple testing error, test a limited number of study characteristics. Although there are no current guidelines for limiting the number of studies, it is prudent to adjust levels of significance according to numbers of comparisons.  **Determine adequate numbers of studies.**  Statistical analyses per subgroup relies on the number of studies included. Too few studies lead to unreliable and unstable estimates. No guidelines restrict threshold numbers for subgroup analyses. However, most reviews use cut-offs of at least 8–10 studies per study characteristic evenly distributed per subgroup (or at least 5 per subgroup).  If the number of studies is too few for dichotomous subgroups, consider meta-regression or restriction analysis. In meta-regression, treat study characteristics (subgroups) as continuous variables using basic regression principles. Restriction analysis involves analyzing specific “homogeneous” studies for estimates.  **Perform meta-analyses per subgroup.**  In Stata, use the by(variable) option in the metan package. Readouts provide subgroup estimates, alongside overall estimates. |

# **Table S9.** Possible database for searching grey literature

| Database | Website | Overview |
| --- | --- | --- |
| Clinical trial registry/Study registries | | |
| - International Clinical Trials Registry Platform | http://apps.who.int/trialsearch/ | A unified system organized by the World Health Organization |
| - ClinicalTrials.gov | https://www.clinicaltrials.gov/ct2/home | Database managed by the National Library of Medicine at the United States National Institutes of Health |
| - EU Clinical Trials Register | https://www.clinicaltrialsregister.eu/ | Database managed by National Competent Authorities and the European Medicines Agency |
| - Cochrane Central Register of Controlled Trials | https://www.cochranelibrary.com/central | Both published and unpublished clinical trials are indexed |
| - ISRCTN registry | https://www.isrctn.com/ | Run by a private institution (International Standard Randomised Controlled Trial Number). This is a preferred partner of the Health Research Agency of the National Health Service (NHS) of the United Kingdom |
| Pre-prints | | |
| - MedRxiv and BioRxiv | <https://www.medrxiv.org/>  https://www.biorxiv.org/ | Jointly managed by the Cold Spring Harbor Laboratory, Yale University, and British Medical Journal |
| - OSF Pre-prints | https://osf.io/preprints/ | Managed by the Center for Open Science – a non-profit organization |
| - Europe PMC | <http://europepmc.org/preprints> | Managed by the European Bioinformatics Institute and partnered/endorsed by PubMed Central. It indexed several pre-print servers, such as, but not limited to arXiv, bioRxiv, medRxiv, PeerJ, ResearchSquare, and SSRN |
| Theses, conference abstracts, and others | | |
| - Google Scholar | https://scholar.google.com/ | Indexing of conference proceedings, thesis and other grey literature (alongside peer-reviewed articles) |

# **Table S10.** Tests for publication selection bias

| Test | Overview | Interpretation | Stata command | R command  library(meta) |  |
| --- | --- | --- | --- | --- | --- |
| Presence of publication selection bias | | | | | |
| Funnel plot | Graphical representation of “missing publication.” It plots the magnitude of the effect estimate (e.g., mean difference, odds ratio) and the standard error. | The large effect estimates are those from smaller studies (larger standard error). Conversely, larger studies (smaller standard error) are those with smaller effect estimates. This plot results to a “funnel” appearance of which the topmost is occupied by large studies with the “most accurate” effect estimate. Any asymmetry is assumed as missing. Given the subjective nature of interpretation, use of the funnel plot without additional formal statistical testing is discouraged. | meta funnelplot  or  metafunnel | funnel() |  |
| Egger’s test | Regression method to detect publication bias | Beta-coefficient is the association between the effect size and the standard error weighted by the inverse variance. A p-value<0.05 where the effect estimates of smaller (less precise) studies do not mimic estimates of larger (more precise) studies may be suggestive of in publication selection bias, but this is neither sensitive nor specific. | meta bias  or  metabias *_ES _seES*, egger | metabias( ,method.bias ="Egger") |  |
| Fail-safe N method | Estimates the number of “null-finding” studies needed for the pooled estimates to be “null.” | It provides an overview of the “robustness” of the pooled effect estimate. A low number relative to the number of studies in the analysis raises a high-risk of publication selection bias. A higher number would mean a more robust result. However, the overall approach is problematic and it tends to give non reliable results. We recommend that it should be avoided.(17) | n.a. | library(metafor)  fsn() |  |
| Adjusting for publication bias | | | | | |
| Trim and fill method | A form of missing data imputation. This graphically fills the missing publication based on the funnel plot. | It provides an adjusted pooled effect estimate if the hypothetical “missing study” exists. The assumption of a perfectly symmetric funnel plot is unrealistic and the method performs very poorly in the presence of heterogeneity.(18, 19) | meta trimfill  or  metatrim | trimmfill() |  |
| Trim and fill extensions (Duval and Tweedle’s method) | An extension of trim and fill using an iterative algorithm using fixed- or random-effects model | It provides an adjusted pooled effect estimate if the hypothetical “missing study” exists | meta trimfill, itermethod(fixed) poolmethod(dlaird)  or  metatrim *_ES _seES*, mixed | trimmfill() |  |

| metan n_tetra mean_tetra sd_tetra n_para mean_para sd_para, nostandard second(random) lcols(Authoryearofpublication n_tetra n_para) texts (100)  Study \| WMD [95% Conf. Interval] % Weight  ---------------------+---------------------------------------------------  Akbal 2013 \| -5.100 -41.858 31.658 0.18  Apstein 1998 \| -6.700 -9.215 -4.185 37.50  Baumann 1992 \|-47.150 -51.862 -42.438 10.68  Brenes 1986 \| -2.340 -29.314 24.634 0.33  Buchholz 2009 \| -6.380 -63.257 50.497 0.07  Cardus 1992 \|-46.300 -55.951 -36.649 2.55  de Groot 2008 \|-23.000 -58.259 12.259 0.19  Farkas 2018 \| 8.940 -46.179 64.059 0.08  Gibson 2008 \| 3.000 -33.114 39.114 0.18  Gorgey 2011A \| -9.000 -36.057 18.057 0.32  Kemp 2000 \| -3.190 -24.349 17.969 0.53  Matos Souza 2010 \| 10.300 0.326 20.274 2.38  Miyatani 2014 \| 1.000 -31.999 33.999 0.22  O Brien 2017 \| 47.240 -3.963 98.443 0.09  Sabour 2013 \|-21.380 -51.006 8.246 0.27  Schmid 2000 \| 33.000 -6.561 72.561 0.15  Schmid 2008 \| 35.000 -15.833 85.833 0.09  Yahiro 2019 \|-22.620 -49.635 4.395 0.32  Wang 2007 \|-17.750 -32.792 -2.708 1.05  Zhong 1995 \|-16.000 -18.374 -13.626 42.07  Laclaustra 2015 \| -9.000 -30.244 12.244 0.53  Wong 2001 \|-23.914 -56.418 8.590 0.22  ---------------------+---------------------------------------------------  I-V pooled WMD \|-15.602 -17.142 -14.062 100.00  D+L pooled WMD \|-10.884 -19.716 -2.053 100.00  ---------------------+---------------------------------------------------  Heterogeneity chi-squared = 307.53 (d.f. = 21) p = 0.000  I-squared (variation in WMD attributable to heterogeneity) = 93.2%  Test of WMD=0 : z= 19.86 p = 0.000 |
| --- |

# **Figure S1.** Sample output on the Stata metan command.

Weighted mean difference of triglyceride levels between individuals with tetraplegia and paraplegia.

We conducted a systematic review of observational studies comparing triglyceride levels (continuous outcome) among different levels of spinal cord injury (exposure) (20). We used the Stata metan package for the analysis. We combined the mean difference among different studies through a random-effects model. We provide sample output (**Figure S1**) and the forest plot (**Figure S2**)

We used a previous meta-analysis on the mean difference in triglyceride levels among individuals with tetraplegia and paraplegia (20). We observed Cochran’s Q *p*-value of <0.001 with an I^2^ of 93.2% (**Figure S1** and **Figure S2**). We categorized as significant heterogeneity, and we interpreted 93.2% heterogeneity as between-study variability, not by chance. In other words, a huge proportion of the variance in association estimates are due to unexplained heterogeneity. We explore one possible source of this heterogeneity later.


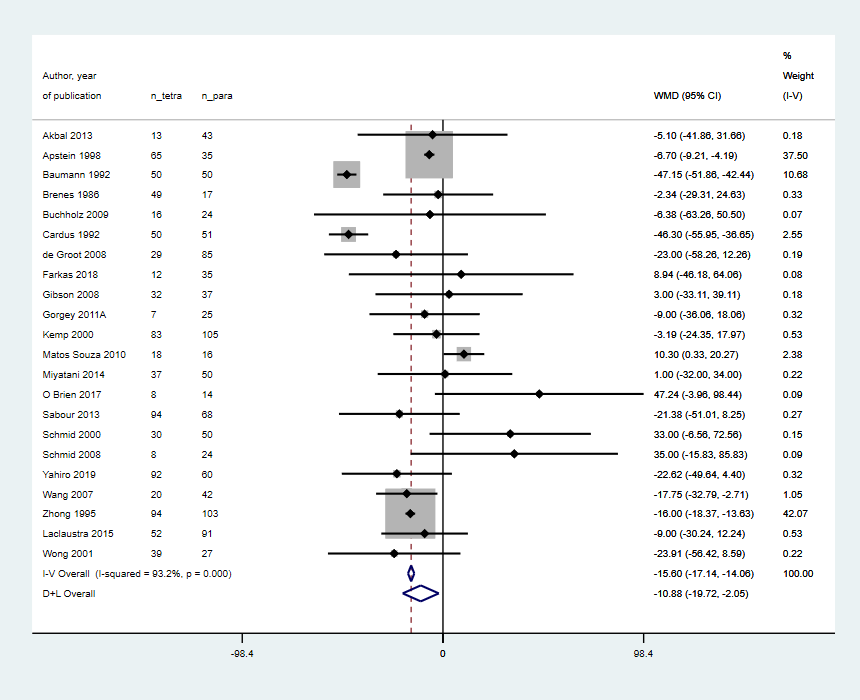


# **Figure S2.** Forest plot on the weighted mean difference of triglyceride levels between individuals with tetraplegia and paraplegia.

Weight assignment and sizes of the box are based on fixed effects.

We conducted a systematic review of observational studies comparing triglyceride levels (continuous outcome) among different levels of spinal cord injury (exposure) (20). We used the Stata metan package for the analysis. We combined the mean difference among different studies through a random-effects model. We provide sample output (**Figure S1**) and the forest plot (**Figure S2**)

We used a previous meta-analysis on the mean difference in triglyceride levels among individuals with tetraplegia and paraplegia (20). We observed Cochran’s Q *p*-value of <0.001 with an I^2^ of 93.2% (**Figure S1** and **Figure S2**). We categorized as significant heterogeneity, and we interpreted 93.2% heterogeneity as between-study variability, not by chance. In other words, a huge proportion of the variance in association estimates are due to unexplained heterogeneity. We explore one possible source of this heterogeneity later.


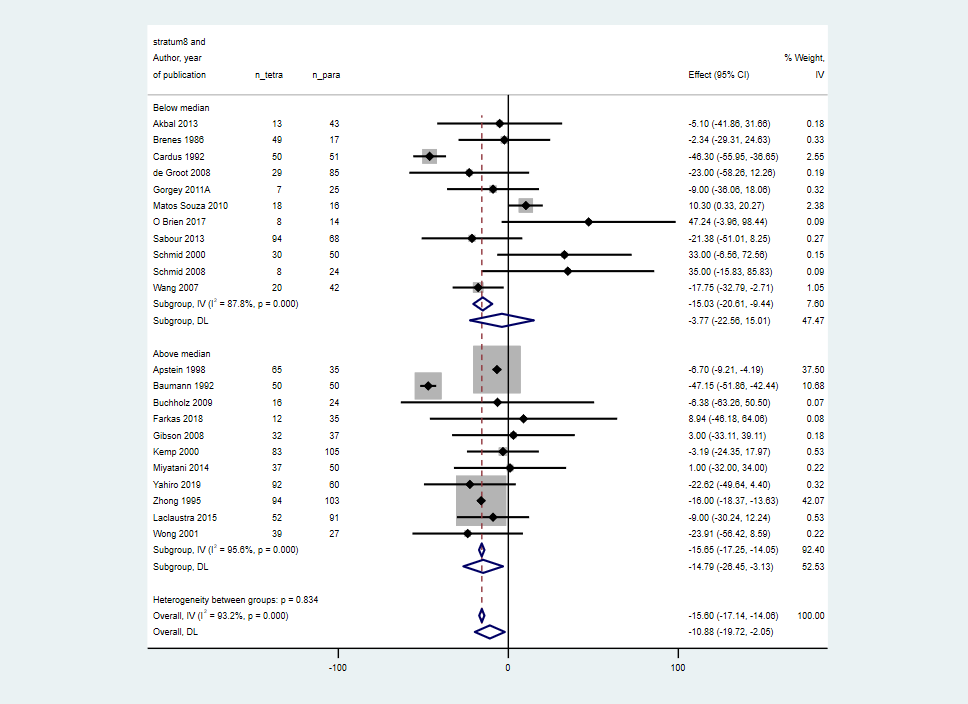


# **Figure S3.** Forest plot on the weighted mean difference of triglyceride levels between individuals with tetraplegia and paraplegia grouped according to age.

Effect estimates and heterogeneity statistics were provided in each subgroup.

In the same triglyceride levels among individuals with tetraplegia vs those with paraplegia example (**Figure S3**) (20), we classified studies according to the mean age of the study population (above and below the overall median age across studies). The heterogeneity for overall analysis was I^2^ 93.2%. Older individuals with tetraplegia had lower serum triglycerides than those with paraplegia (WMD -14.8 mg/dL, 95% CI -26.5, -3.1), albeit with high heterogeneity (I^2^ = 95.6%). We did not observe this change when comparing younger individuals with tetraplegia and paraplegia. We cautiously concluded that age could be a factor impacting triglyceride differences we found between people with tetraplegia and paraplegia. However, keep in mind the calculation’s power (22 studies), the cross-sectional nature of the observation (snapshot of each study), the unadjusted estimates, and the kind of data—study-level, not participant-level data—we used (thus, we could not exclude an ecological fallacy). With the presence of comorbidities and/or etiology of tetraplegia and paraplegia, age can also be related, which could contribute to our observed results (see ecological fallacy in section 6.3). Remember, such analyses are exploratory to generate hypotheses tested in future studies.


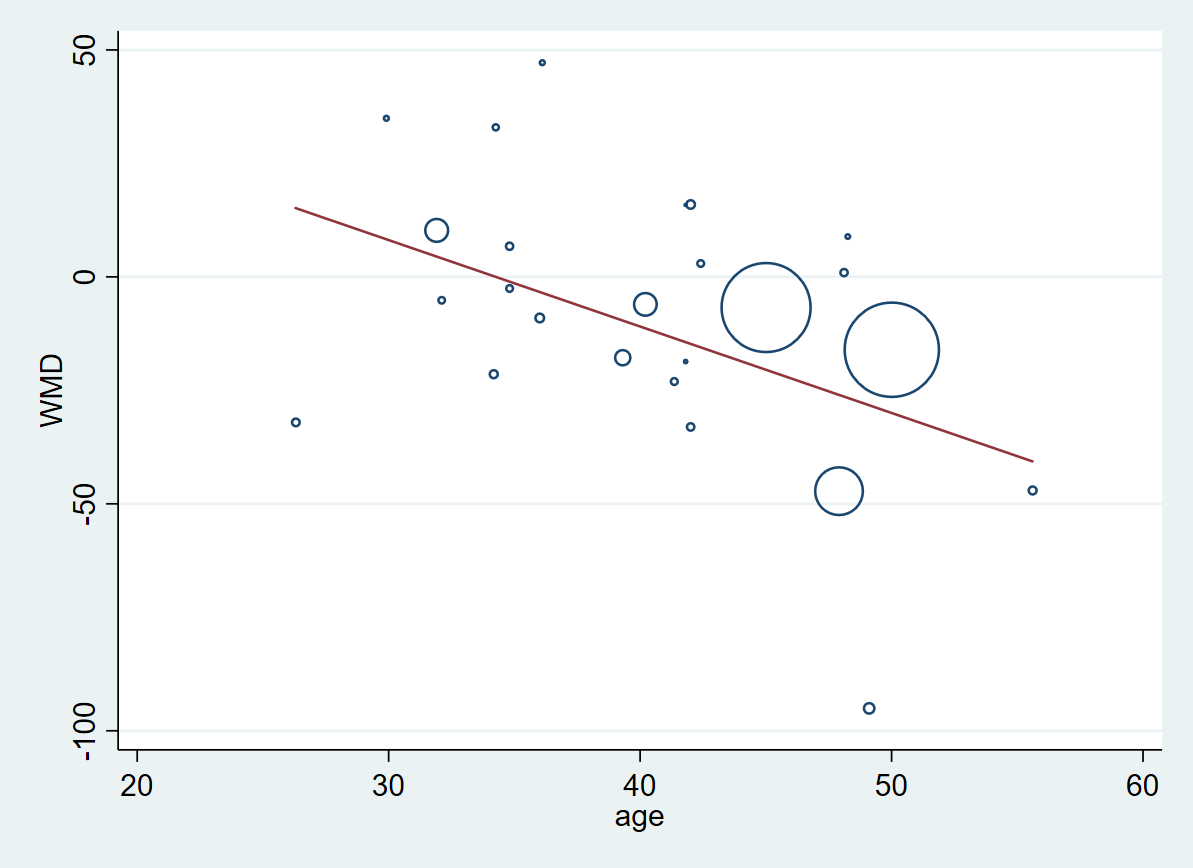


**Figure S4.** Bubble plot showing the weighted mean difference between the triglyceride levels of tetraplegia versus paraplegia (y-axis) and the study level mean age.

Meta-regression Number of obs = 22

REML estimate of between-study variance tau2 = 336.1

% residual variation due to heterogeneity I-squared_res = 93.43%

Proportion of between-study variance explained Adj R-squared = -5.97%

With Knapp-Hartung modification

------------------------------------------------------------------------------

_ES | Coef. Std. Err. t P>|t| [95% Conf. Interval]

-------------+----------------------------------------------------------------

age | -.4814112 .6102925 -0.79 0.439 -1.754459 .7916366

_cons | 9.243163 25.21815 0.37 0.718 -43.36098 61.8473

------------------------------------------------------------------------------

The circles represent each study, and the size corresponds to the weight assigned to the study. The line represents the best-fitted regression line considering the weighted effect estimates of each study. Also shown is the readout using Stata metareg.

| ------------------------------------------------------------------------------  Study omitted \| Estimate [95% Conf. Interval]  -------------------+----------------------------------------------------------  Akbal 2013 \| -11.065914 -20.065336 -2.066493  Apstein 1998 \| -10.042924 -20.693733 .60788465  Baumann 1992 \| -9.5635958 -16.525101 -2.6020916  Brenes 1986 \| -11.266644 -20.324043 -2.2092445  Buchholz 2009 \| -10.95646 -19.884584 -2.0283365  Cardus 1992 \| -8.4073792 -17.280897 .46613899  de Groot 2008 \| -10.431992 -19.441301 -1.4226813  Farkas 2018 \| -11.267333 -20.192595 -2.3420699  Gibson 2008 \| -11.358391 -20.353527 -2.3632531  Gorgey 2011A \| -10.947528 -20.012003 -1.8830528  Kemp 2000 \| -11.291865 -20.398449 -2.1852815  Matos Souza 2010 \| -12.623569 -21.642218 -3.6049209  Miyatani 2014 \| -11.330317 -20.344278 -2.3163552  O Brien 2017 \| -12.19021 -21.071009 -3.3094115  Sabour 2013 \| -10.409785 -19.455591 -1.3639792  Schmid 2000 \| -12.283833 -21.20838 -3.3592873  Schmid 2008 \| -11.918666 -20.822411 -3.0149224  Yahiro 2019 \| -10.309785 -19.37426 -1.2453084  Wang 2007 \| -10.358358 -19.55711 -1.1596075  Zhong 1995 \| -8.5880995 -20.632072 3.4558744  Laclaustra 2015 \| -10.955361 -20.0716 -1.8391242  Wong 2001 \| -10.354497 -19.379566 -1.3294288  -------------------+----------------------------------------------------------  Combined \| -10.884231 -19.715812 -2.0526503  ------------------------------------------------------------------------------  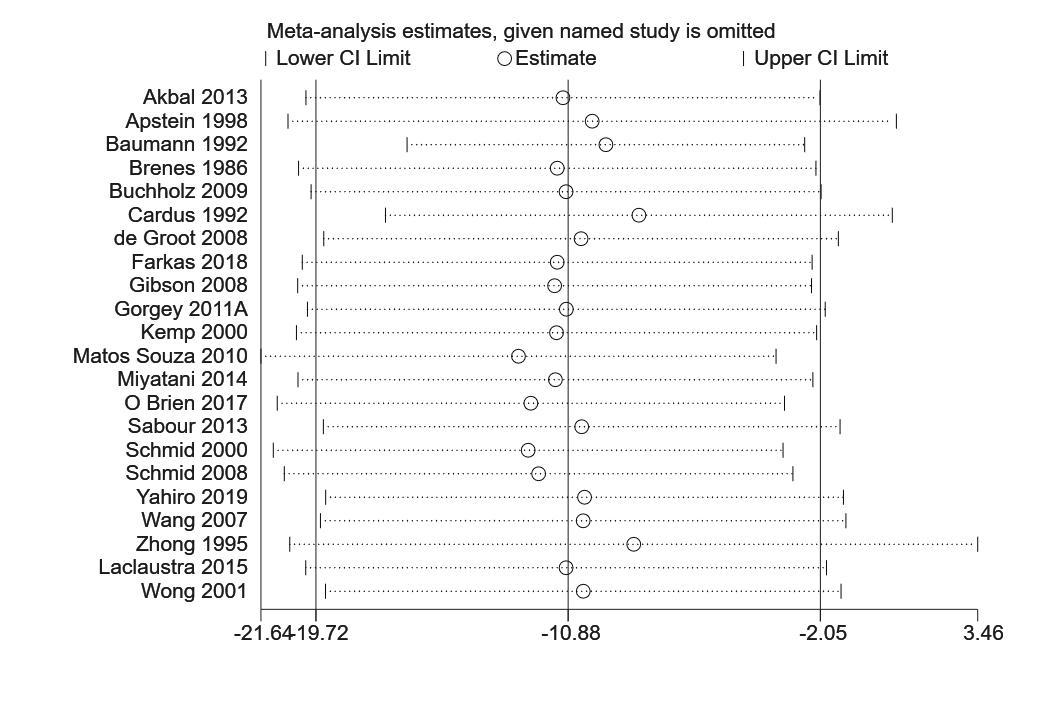 |  |
| --- | --- |

# **Figure S5.** Graph and test readout from Stata metaninf package on leave-one out analysis using the comparison of triglyceride levels between tetraplegia and paraplegia.

In the graph, the horizontal axis shows the effect estimates while the vertical axis shows the study. The bars in the graph show the effect estimates (circle) and its confidence intervals (upper limit and lower limit) upon the removal of the study indicated in the vertical axis. The readout below shows the exact values.

In the same study about triglyceride levels among individuals with tetraplegia vs those with paraplegia, (20) we showed the recomputed association estimate after omitting one study at a time (**Figure S5**). We identified three studies (Apstein, 1998; Cardus, 1992; and Zhong, 1995) that pushed the summary association estimate to null (**Figure S5**). The plot showed how CIs changed when removing one study at a time. Apstein 1998 and Zhong 1995 made the summary association estimate wider and towards null. In the next step, review the study characteristics of the identified influential study to investigate differences from other studies.


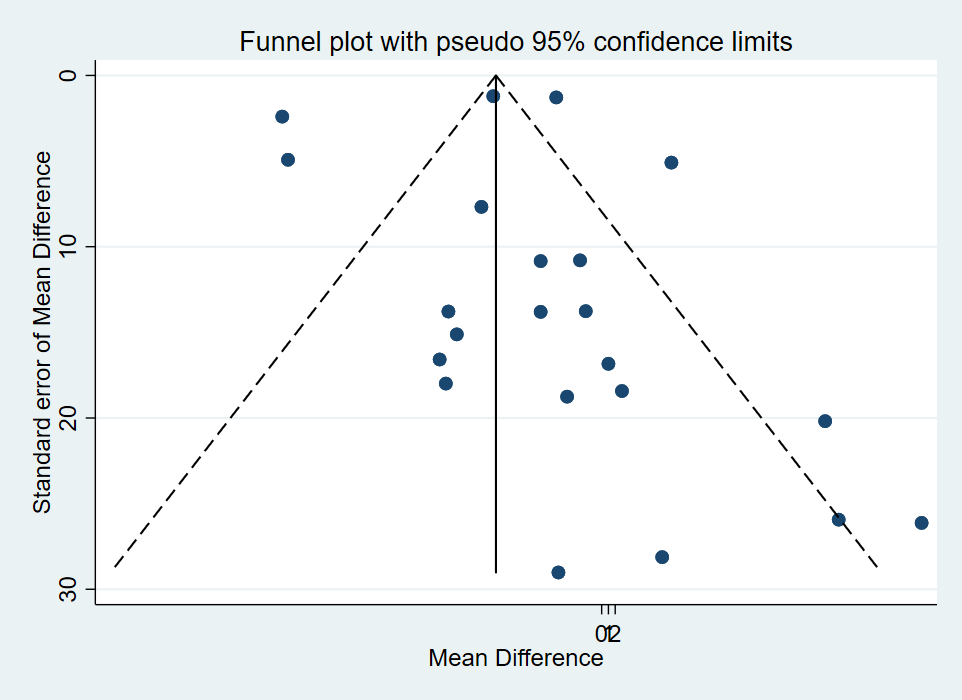


# **Figure S6.** Funnel plot of studies comparing the triglyceride levels of tetraplegia and paraplegia.

The horizontal axis shows the triglyceride mean difference of the two groups (effect size), while the vertical axis shows the standard error (precision). Each dot represents a study effect estimate. The broken lines represent the computed pseudo 95% confidence limits, while the overall mean difference is depicted by the solid vertical line

Using the same study about triglyceride levels among individuals with tetraplegia vs those with paraplegia, (20) we made a funnel plot (**Figure S6**) with metafunnel, then tested for small-study effects with the metabias package in Stata (21). **Figure S7** is a sample readout using Egger’s regression. The intercept or bias is 0.274 with a *p*-value of 0.792. Thus, our pooled analysis—given Egger’s test is accurate—showed no indication for small study effects.

Egger's test for small-study effects:

Regress standard normal deviate of intervention

effect estimate against its standard error

Number of studies = 22 Root MSE = 3.914

------------------------------------------------------------------------------

Std_Eff | Coef. Std. Err. t P>|t| [95% Conf. Interval]

-------------+----------------------------------------------------------------

slope | -16.19584 3.795499 -4.27 0.000 -24.11311 -8.278567

bias | .2749208 1.029958 0.27 0.792 -1.873534 2.423376

------------------------------------------------------------------------------

Test of H0: no small-study effects P = 0.792

# **Figure S7.** Read-out of the test for small study effects (Egger’s regression) from Stata using metabias.

Using the same study about triglyceride levels among individuals with tetraplegia vs those with paraplegia, (20) we made a funnel plot (**Figure S6**) with metafunnel, then tested for small-study effects with the metabias package in Stata (21). **Figure S7** is a sample readout using Egger’s regression. The intercept or bias is 0.274 with a *p*-value of 0.792. Thus, our pooled analysis—given Egger’s test is accurate—showed no indication for small study effects.

# **References**

1. Westreich D. Berkson's bias, selection bias, and missing data. Epidemiology (Cambridge, Mass). 2012;23(1):159-64.

2. Guthrie KA, Sheppard L. Overcoming biases and misconceptions in ecological studies. Journal of the Royal Statistical Society: Series A (Statistics in Society). 2001;164(1):141-54.

3. Wang Y, Pirani M, Hansell AL, Richardson S, Blangiardo M. Using ecological propensity score to adjust for missing confounders in small area studies. Biostatistics. 2019;20(1):1-16.

4. Viswanathan M, Berkman ND, Dryden DM, Hartling L. AHRQ Methods for Effective Health Care. Assessing Risk of Bias and Confounding in Observational Studies of Interventions or Exposures: Further Development of the RTI Item Bank. Rockville (MD): Agency for Healthcare Research and Quality (US); 2013.

5. Programme CAS. CASP Checklist2018 2 April 2021. Available from: <https://casp-uk.net/casp-tools-checklists/>.

6. Moola S MZ, Tufanaru C, Aromataris E, Sears K, Sfetcu R, Currie M, Qureshi R, Mattis P, Lisy K, Mu P-F. . Chapter 7: Systematic reviews of etiology and risk. JBI Manual for Evidence Synthesis2020.

7. Health NIo. Quality Assessment Tool for Observational Cohort and Cross-Sectional Studies.: National Institutes of Health; 2014.

8. Wells GA, Shea B, O’Connell Da, Peterson J, Welch V, Losos M, et al. The Newcastle-Ottawa Scale (NOS) for assessing the quality of nonrandomised studies in meta-analyses. Oxford; 2000.

9. Arjola Bano LC, Taulant Muka, Francesco U.S. Mattace-Raso, Lia Bally, Oscar H. Franco, Robin P. Peeters, and Salman Razvi. Thyroid Function and the Risk of Fibrosis of the Liver, Heart, and Lung in Humans: A Systematic Review and Meta-Analysis. Thyroid. 2020;30(6):806-20.

10. Modesti PA, Reboldi G, Cappuccio FP, Agyemang C, Remuzzi G, Rapi S, et al. Panethnic Differences in Blood Pressure in Europe: A Systematic Review and Meta-Analysis. PLOS ONE. 2016;11(1):e0147601.

11. Sterne JA, Hernan MA, Reeves BC, Savovic J, Berkman ND, Viswanathan M, et al. ROBINS-I: a tool for assessing risk of bias in non-randomised studies of interventions. BMJ. 2016;355:i4919.

12. Rothman KJ, Greenland S, Lash TL. Modern Epidemiology. 3rd ed. *Philadelphia, PA*: *Lippincott, Williams & Wilkins*; 2008.

13. Westreich D. Epidemiology by Design: A Causal Approach to the Health Sciences. Oxford University Press; 2020.

14. Muka T, Glisic M, Milic J, Verhoog S, Bohlius J, Bramer W, et al. A 24-step guide on how to design, conduct, and successfully publish a systematic review and meta-analysis in medical research. European journal of epidemiology. 2020;35(1):49-60.

15. Li T, Higgins JP, Deeks JJ. Collecting data. Cochrane handbook for systematic reviews of interventions. 2019:109-41.

16. Li T, Vedula SS, Hadar N, Parkin C, Lau J, Dickersin K. Innovations in data collection, management, and archiving for systematic reviews. Annals of internal medicine. 2015;162(4):287-94.

17. Becker BJ. Failsafe N or File-Drawer Number. Publication Bias in Meta‐Analysis2005. p. 111-25.

18. Terrin N, Schmid CH, Lau J, Olkin I. Adjusting for publication bias in the presence of heterogeneity. Stat Med. 2003;22(13):2113-26.

19. Peters JL, Sutton AJ, Jones DR, Abrams KR, Rushton L. Performance of the trim and fill method in the presence of publication bias and between-study heterogeneity. Stat Med. 2007;26(25):4544-62.

20. Raguindin PF, Frankl G, Itodo OA, Bertolo A, Zeh RM, Capossela S, et al. The neurological level of spinal cord injury and cardiovascular risk factors: a systematic review and meta-analysis. Spinal cord. 2021;59(11):1135-45.

21. Harbord RM, Harris RJ, Sterne JA. Updated tests for small-study effects in meta-analyses. Stata J. 2009;9(2):197-210.
